# Ossification of the Ligamentum Flavum in a Nineteenth-Century Skeletal Population Sample from Ireland: Using Bioarchaeology to Reveal a Neglected Spine Pathology

**DOI:** 10.1038/s41598-018-27522-x

**Published:** 2018-06-18

**Authors:** Jonny Geber, Niels Hammer

**Affiliations:** 1 Department of Anatomy, University of Otago, Dunedin, New Zealand; 2 Department of Orthopaedic and Trauma Surgery, University of Leipzig, Leipzig, Germany

## Abstract

Ossification of the ligamentum flavum of the spine (OLF) is rarely reported in individuals of European ancestry. It has, however, been observed in archaeological skeletons from Europe. The aim of this study was to revisit OLF rates, utilising a mid-nineteenth-century skeletal sample from Ireland. The hypothesis was that the OLF prevalence rate was similar to studies on non-Europeans. Spines from 345 individuals were analysed, and the extent of OLF recorded on the cranial and caudal attachment sites of each vertebra using the following classification system: Grade 0 (no change); Grade 1 (<2 mm); Grade 2 (2–4 mm); Grade 3 (≥4 mm). OLF was observed at prevalence rates of 83.6%. There was no disparity in prevalence based on sex, except for individuals aged 36–45 years at death in which the male rate was higher. Advancing age was a determining factor in the OLF occurrence with an onset in young adulthood (18–25 years), and most severe grades occurring over the age of 25 years. OLF coincides with spinal osteoarthritis, spondylosis deformans and Schmorl’s nodes in both sexes, and with intervertebral osteochondrosis in females. The results of this study indicate that OLF is likely to be an understudied rather than rare condition in European populations.

## Introduction

Ossification of the ligamentum flavum (OLF) is a spinal pathology that is currently most frequently clinically reported in Japanese and Chinese populations^1–32^. In those, the prevalence of OLF lay within the range of 3.8% to 26.0%^3,16^, with males being more affected clinically than females^2^ and increasing in rate with advancing age^6,9^. Myelopathy is the OLF-defining pathology with subsequent morbidity. The lower thoracic spine is the most commonly affected region^2,9^. Cervical and lumbar manifestations have also been described^33,34^, and single-level ossification is reported more often than multi-level manifestation^6^. Both intrinsic and extrinsic factors have been postulated as the cause of OLF. Intrinsic factors include genetic predisposition, dietary factors with related obesity and type 2 diabetes. Extrinsic factors are in general attributed to an altered mechanical load of the spine resulting in trauma^29^. Some overlap appears to exist between both^35^.

The baseline data for the aetiology, diagnosis, treatment and long-term outcomes of the condition are, however, inconsistent. Moreover, based on the published clinical literature, OLF appears to be exceptionally rare in populations with predominately European ancestry. The reported cases are almost exclusively based on observations in East-Asian individuals, which conflicts the theory of OLF being a major effect of altered biomechanics and physical activity. Vice versa, one may speculate that OLF is a neglected condition in individuals of European ancestry, e.g. due to a more predominant focus on clinical and radiological pathologies such as hip and knee arthritis and peripheral vascular disease. Spinal ligament ossifications are a common observation in bioarchaeological samples^36^, and OLF is of particular interest in these as it is present on the in general best surviving portion of human vertebrae from archaeological contexts and therefore usually available for study. While OLF has been reported in archaeological skeletons from Europe, these observations have however rarely been published^37–39^.

**Figure 1:**
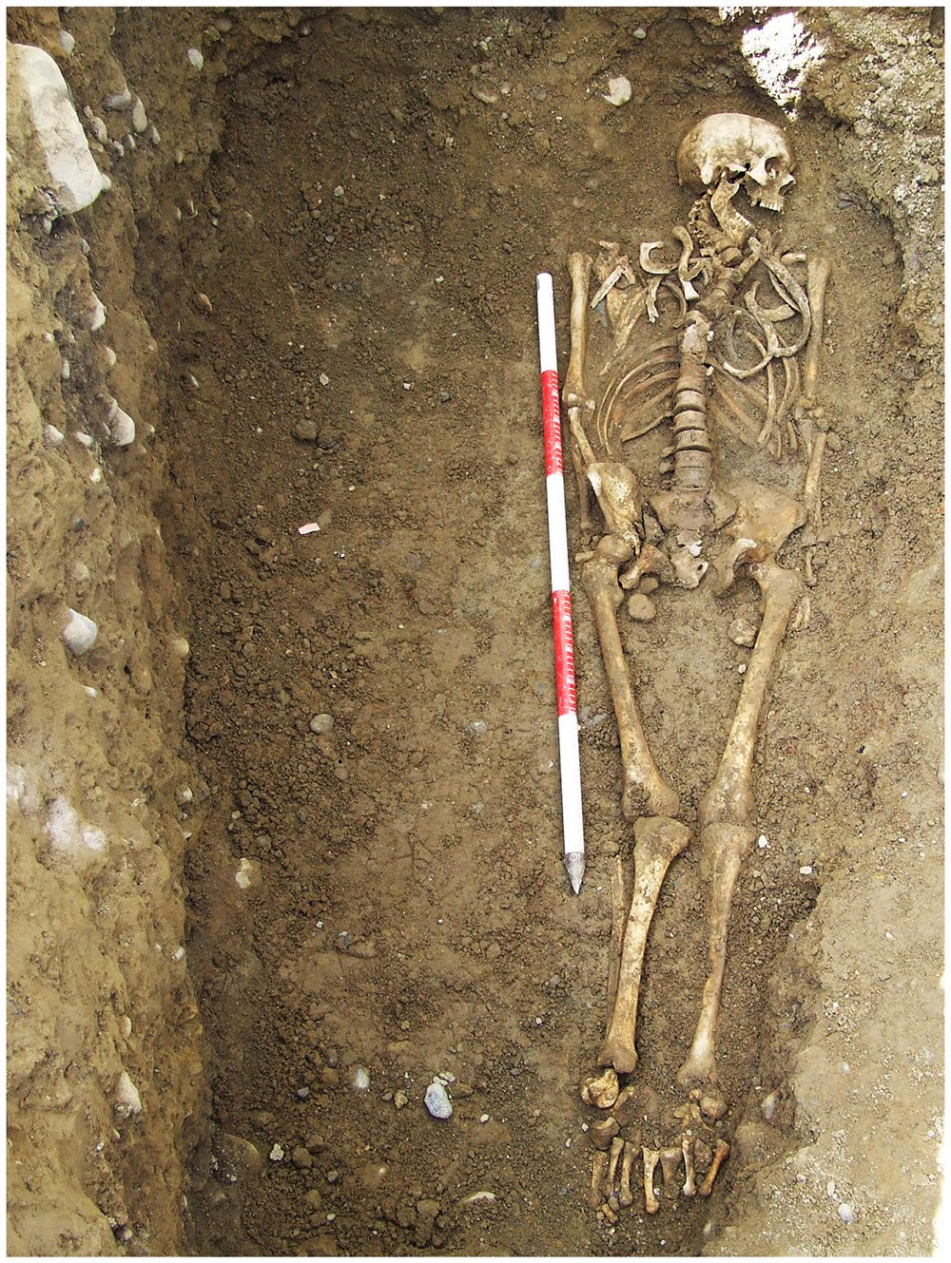
*In-situ* photograph of an adult male skeleton (36–45 years old) from the Kilkenny Union Workhouse cemetery. Photo: Margaret Gowen & Co. Ltd.

This article is reporting on OLF in archaeological skeletal remains (Fig. 1) from a mid-nineteenth-century (AD 1847–1851) population sample from Kilkenny City, Ireland, and the prevalence and frequency rates are discussed in conjunction with clinical reports of OLF. The aim of this study is to highlight the value of bioarchaeological data when discussing the epidemiology of the condition today. The study provides further evidence from the palaeopathological record that OLF may be an underreported pathological condition in individuals of European ancestry. The material comprises of remains of people from the lower social strata that died as a consequence of malnutrition and infectious disease in a workhouse during the notorious Great Irish Famine between 1845 and 1852^40^. Using the skeletal sample from Kilkenny, the existence of OLF is quantified, using a macroscopic recording methodology adapted for archaeological human remains from which the following hypotheses are formed:

(A) Ossification of the ligamentum flavum is present in a skeletal sample of a nineteenth-century population of European (Irish) ancestry and is occurring at rates and frequencies that are comparable to those reported in the clinical literature on Japanese and Chinese populations.
(B) Ossification of the ligamentum flavum progresses in rates, frequency and severity with age, and is more frequent in males than in females.

## Material and Methods

The given sample comprised of skeletons of individuals who had died in the Union Workhouse in Kilkenny City, Ireland, during the Great Irish Famine between August 1847 and March 1851^40^. These individuals belonged to the lowest strata of contemporaneous society, and they maintained their subsistence in dire socioeconomic circumstances while relying on a monoculture economy that was largely dependent on the potato crop. The primary cause of the Famine was a potato blight^41^ and, as a consequence, hundreds of thousands of people in Ireland resorted to the workhouses for relief as a mean to avoid death by starvation^42^. Of the over 4,100 people who died in the Kilkenny Union Workhouse during the Famine^43^, at least 970 individuals were buried in an unmarked intramural mass burial ground. This burial ground was subject to a development-driven archaeological excavation in 2006, from which the human remains were subsequently analysed and studied before to their final re-interment in 2010 (see below). While their demise is related to starvation and malnutrition, the primary direct causes of their deaths would have been infectious diseases such as typhus, typhoid fever, cholera, dysentery and tuberculosis^40^. The analysed population sample comprised of individuals who at the time of their deaths were dependent on poor relief^44^. No historical records survive that can shed light on the identity of these people, but a broad contextualisation of the social fabric of Kilkenny City during this period can tell us that they—prior to institutionalisation—would have been servants, agricultural workers and cottiers, industrial workers, as well as tradesmen and those who were deemed completely destitute^40^.

Following excavation, the human remains were cleaned in lukewarm water and subsequently air-dried. The skeletons were analysed by J.G. following a standard osteological protocol^45,46^. Sex was determined from cranial and pelvic morphology^47^. Age-at-death was estimated from cranial suture obliteration, the morphology of the pubic symphyses, the coxal bones’ auricular surfaces and sternal ends of ribs^48–52^. Only individuals that could be assigned a sex and an estimated age range were included in this study. Age estimations from human skeletal remains determine the biological or skeletal age and is fundamentally different from the true chronological age of an individual^53^. Direct analogies with clinical data, based on skeletal age estimates, are therefore not encouraged; instead, a comparative analysis should focus on age-related trends and patterns. Bone preservation of each skeleton was assessed using the system devised by McKinley^54^.

All the skeletal remains were reinterred at the Famine Memorial Garden at the MacDonagh Junction complex, Hebron Road, Kilkenny City, Ireland, in 2010. Bioarchaeological analyses of the human remains from the Kilkenny Union Workhouse have been undertaken by licence (05E0435) from the National Museum of Ireland.

### Macroscopic assessment, recording and quantification of OLF

**Figure 2:**
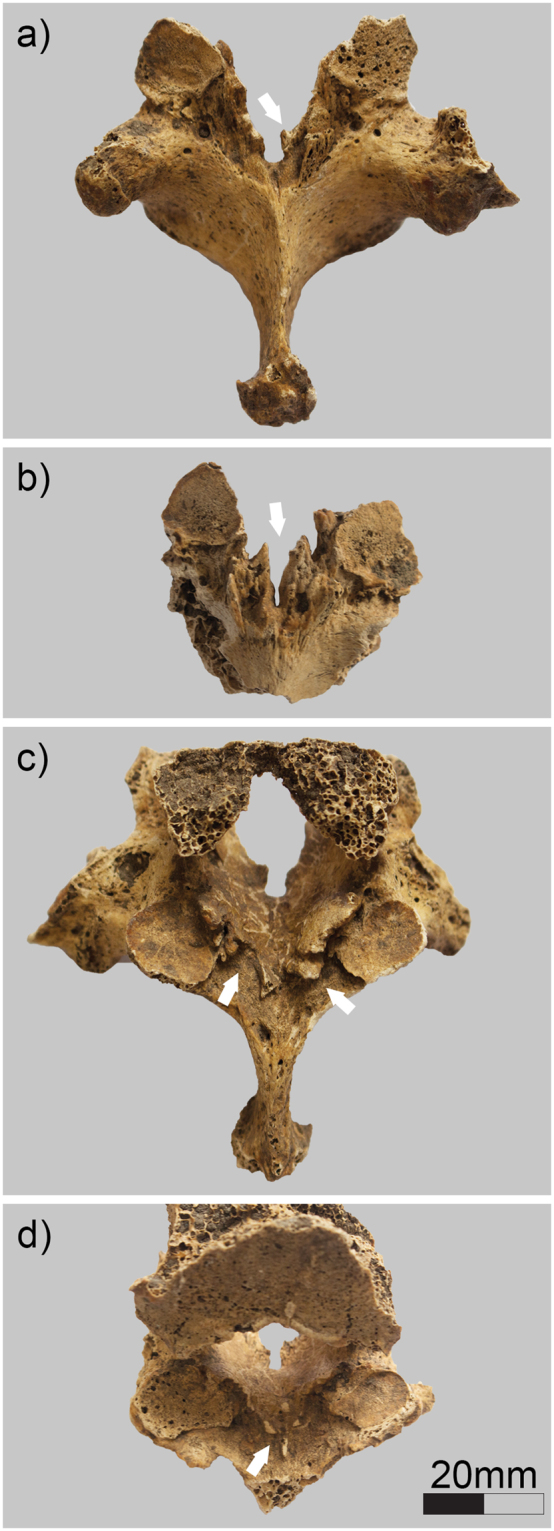
Examples of ossification of the ligamentum flavum in archaeological human vertebrae (adult male), involving the cranial (**a** = T9: Grade 2; **b** = T6: Grade 3) and caudal (**c** = T9: Grade 3; **d** = T8: Grade 1) attachments. Arrows indicate the ossifications.

The presence of OLF was assessed and recorded at the cranial and caudal attachments of the vertebral arches^38,55,56^ (Fig. 2). The occurrence of OLF was assessed both by crude prevalence rates per spines and by frequencies by vertebral level. When recorded by level, the junction between two vertebrae was considered (e.g. C7/T1, T7/T8, L2/L3). The more developed ossification (if present) of either the cranial or caudal attachment of any of two adjacent vertebrae was scored. In total, 2,887 levels were assessed in male skeletons, and 2,696 levels in female skeletons. Due to the incompleteness of the majority of the given vertebral columns, OLF rates per spines were calculated on the basis of relative completeness. A spine was considered observable when at least the thoracic region was present. Due to the incomplete nature of archaeological human skeletons (of the 345 individuals included in this study, only two skeletons display vertebral columns that are 100% preserved), the thoracic region was recorded as observable if at least six thoracic vertebrae were present, and a lumbar region if at least three lumbar vertebrae were present, following accepted praxis^57^.

### Statistical analyses

SPSS 24.0 software (IBM, IL, USA) and Excel 2013 (Microsoft Corporation, Redmond, WA, USA) were used to evaluate the data statistically. The Kolmogorov-Smirnov test was used to determine normal distribution. Chi-square tests were employed when comparing prevalence rates and conducting correlation analyses, and a binary logistic regression analysis was used to determine the relationship of age group and sex to the presence of OLF.

### Ethics statement

The tissues are classified as ‘archaeological artefacts’ according to the National Monuments Act in Ireland, and consequently, no ethical approvals was required for the described study.

## Results

### Taphonomic factors do not affect OLF rates in this skeletal sample

There was a total of 2,268 cervical, 3,614 thoracic and 1,493 lumbar vertebrae from 425 adults (≥18 years) available for this study. The least complete spines were present in ≥46-year-old females and 36 to 45-year-old males (Table 1), although no statistical significance in difference was observed. The average number of vertebrae present per skeleton was closely related to bone preservation, where the best-preserved skeletons (Grade 0 = excellent) exhibited an average of 6.5 cervical, 11.6 thoracic and 4.8 lumbar vertebrae. The least-preserved skeletons (Grade 5 = very poor) exhibited an average of 5.7 cervical vertebrae, 6.8 thoracic and 3.8 lumbar vertebrae (χ^2^(120) = 251.0, *p* < 0.001). Completeness of each skeleton was due to truncation and other post-depositional disturbances of the remains. As a consequence, the proportion included in this study was 88.6% (179/202) of the males and 89.8% (166/185) of the females. This implies that the general representation of OLF, as observed macroscopically from the osteological analysis of the remains, is close to a true reflection of the condition in the total population sample.

**Table 1:** Quantitative data of the number of observable vertebrae and relative completeness of spines in adult skeletons from the Kilkenny Union Workhouse, by sex and age group.

Sex/Age group (years)	MNI	Cervical		Thoracic		Lumbar	
		N	N:MNI	N	N:MNI	N	N:MNI
**Males**							
18–25	18	108	6.0	197	10.9	82	4.6
26–35	46	278	6.0	446	9.7	181	3.9
36–45	94	516	5.5	833	8.9	352	3.7
≥46	44	274	6.2	388	8.8	158	3.6
Total	202	1,176	5.8	1,864	9.2	773	3.8
**Females**							
18–25	17	109	6.4	189	11.1	79	4.6
26–35	74	411	5.6	668	9.0	275	3.7
36–45	74	457	6.2	718	9.7	297	4.0
≥46	20	115	5.8	175	8.8	69	3.5
Total	185	1,092	5.9	1,750	9.5	720	3.9

MNI = Minimum Number of Individuals.

### OLF is present in over 80% of adults from a mid-nineteenth-century Irish population sample

The analysis revealed high rates of OLF in the thoracolumbar spines of the Kilkenny sample. The total prevalence of was 83.6% (272/345), with 83.2% (273/328) of thoracic vertebral columns and 28.6% (88/308) of lumbar spines affected. When quantified by vertebrae counts, 39.2% of thoracic (1,417/3,614) and 3.3% of lumbar (49/1,493) vertebrae (either on the cranial or caudal portion) were displaying OLF to some degree. The condition was not observed in any cervical vertebrae (0/2,268) in this sample. The extent of the ossifications was classified by grade of severity (Table 2). Slight ossifications (Grade 1) were the most common manifestations and were observed in 75.9% (262/345) of the total sample. Moderate ossifications (Grade 2) were present in 47.5% (164/345), and considerable ossifications (Grade 3) in 12.8% (44/345).

**Table 2:** Classification for scoring the grade of ossification of the ligamentum flavum (by length/dimension of ossifications) in vertebrae.

Grade	Description
0	No visible change
1	<2 mm
2	2–4 mm
3	≥4 mm

**Figure 3:**
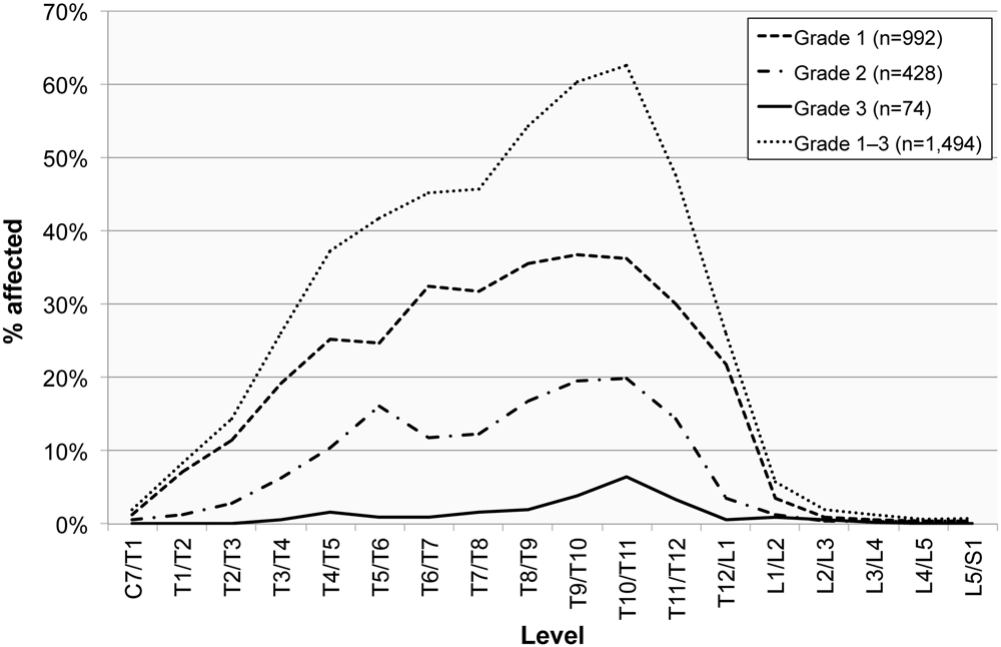
The frequency of ossification of the ligamentum flavum manifested by spinal level in adult males (n = 179) and females (n = 166), by the degree of ossification (Grade 1 = slight, Grade 2 = moderate; Grade 3 = considerable).

The highest frequencies in this sample occurred at the T10/T11 level (36.2%; 111/307), and there were two further peaks apparent at the T6/T7 (32.5%; 102/314) and the T11/T12 (30.0%; 92/307) levels. Grade 1 ossifications displayed a peak in frequency at the T9/T10 level (36.8%; 113/307). This pattern was different for the most pronounced ossifications, which were primarily noticeable one level down at T10/T11 (Grade 2 = 19.9%; 61/307; Grade 3 = 6.5%; 20/307) (Fig. 3).

The high rates of OLF in this population sample, and of slight ossifications (Grade 1) in particular, does warrant the question on whether the majority of these skeletal changes should be considered a normal variation, or a pathological condition. Grade 1 OLF, as recorded macroscopically in this study, is unlikely to be detected through radiographic examinations in clinical cases. Their relevance as indicators of pathological (i.e. symptomatic) conditions is therefore difficult to ascertain in this study and would require an autopsy sample to either confirm or reject such a hypothesis.

### The number of levels affected depends on the grade of ossification

**Figure 4:**
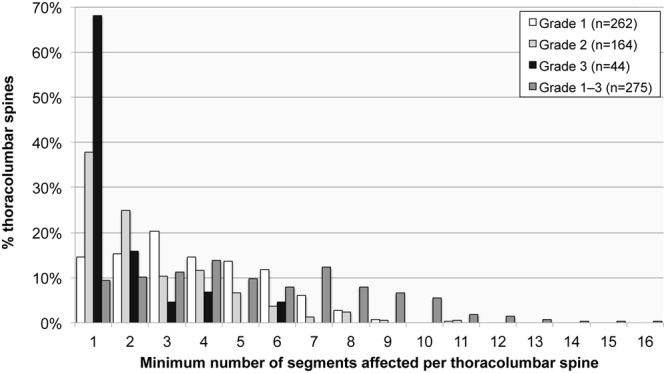
The minimum number of levels affected by of ossification of the ligamentum flavum in thoracolumbar spines, irrespective of anatomical completeness of vertebral columns, by the degree of ossification.

The minimum number of levels affected by OLF in individual spines was, in general, less than five and ranged from one to 16 (Fig. 4). As is usually the case with archaeological skeletons, however, the anatomical completeness of the spines differed between individuals (see the Methodology section below). Of the 231 individuals (117 males and 114 females) with complete thoracolumbar spines, the number of affected levels of those with OLF (Grade 1–3) (n = 191) ranged from one to 16 ($\bar{x}$ = 6.1; SD = 3.1).

The number of levels affected by Grade 1 OLF ranged from one to 11 ($\bar{x}$ = 4.1; SD = 2.1), with the highest proportion of individuals (19.0%; 35/184) being affected at three levels. A different pattern was evident when only considering Grade 2 or Grade 3 manifestations, which was more consistent with the reports in the clinical literature^6^. In individuals with Grade 2 OLF present, the number of levels affected ranged from one to 11 ($\bar{x}$ = 2.9; SD = 2.1), with the highest percentage (33.3%; 39/117) being single-level involvement. In thoracolumbar spines with Grade 3 OLF, the range stretched from one to six ($\bar{x}$ = 1.6; SD = 1.2), with 69.4% (25/36) of all cases being single-level ossifications.

### There is no clear disparity in OLF prevalence observed between females and males

Males in the given sample displayed higher OLF rates than females, with a ratio of 1.1:1 in thoracic spines and 1.3:1 in lumbar spines (Table 3). A statistically significant difference was found between males and females in OLF prevalence rates within the 36 to 45-year-old age group (χ^2^(1) = 4.2, *p* = 0.04). This difference is likely to be a methodological error relating to the difficulties in determining the age-at-death in post-pubertal human skeletons and adults of over the age of 30 years in particular^53^. A binary logistic regression analysis, however, indicated that on an overall level, sex did not influence the presence or non-presence of OLF in this population (Table 4).

**Table 3:** Prevalence rate and frequency [square brackets] of ossification of the ligamentum flavum in adult males and females (df = 1).

Region	Males		Females		χ^2^	*p*-value
	n/N	%	n/N	%		
Cervical	0/169 [0/1,176]	0.0 [0.0]	0/160 [0/1,092]	0.0 [0.0]	—	—
Thoracic	150/171 [790/1,864]	87.7 [42.4]	123/157 [627/1,750]	78.3 [35.8]	5.2	0.023
Lumbar	51/161 [29/773]	31.7 [3.8]	37/147 [20/720]	25.2 [2.8]	1.6	0.207
Thoracolumbar	150/171 [819/2,637]	87.7 [31.1]	125/158 [647/2,470]	79.1 [26.2]	4.4	0.035

**Table 4:** Binary logistic regression values of ossification of the ligamentum flavum for vertebral region by sex (female = 1; male = 2) and age group.

Region	Predictor	Coeff (SE)	Odds ratio (CI)	*p*-value
Thoracic	Constant	−5.9 (1.4)	—	<0.001
	Sex	0.5 (0.3)	1.7 (0.9, 3.2)	0.090
	Age group	0.9 (0.2)	2.5 (1.7, 3.6)	<0.001
Lumbar	Constant	−2.0 (1.2)	—	0.077
	Sex	0.3 (0.3)	1.4 (0.8, 2.2)	0.247
	Age group	0.1 (0.2)	1.1 (0.8, 1.6)	0.557
Thoracolumbar	Constant	−6.0 (1.5)	—	<0.001
	Sex	0.5 (0.3)	1.6 (0.9, 3.0)	0.125
	Age group	0.9 (0.2)	1.5 (1.7, 3.7)	<0.001

CI = confidence interval, Coeff = coefficient, SE = standard error.

**Figure 5:**
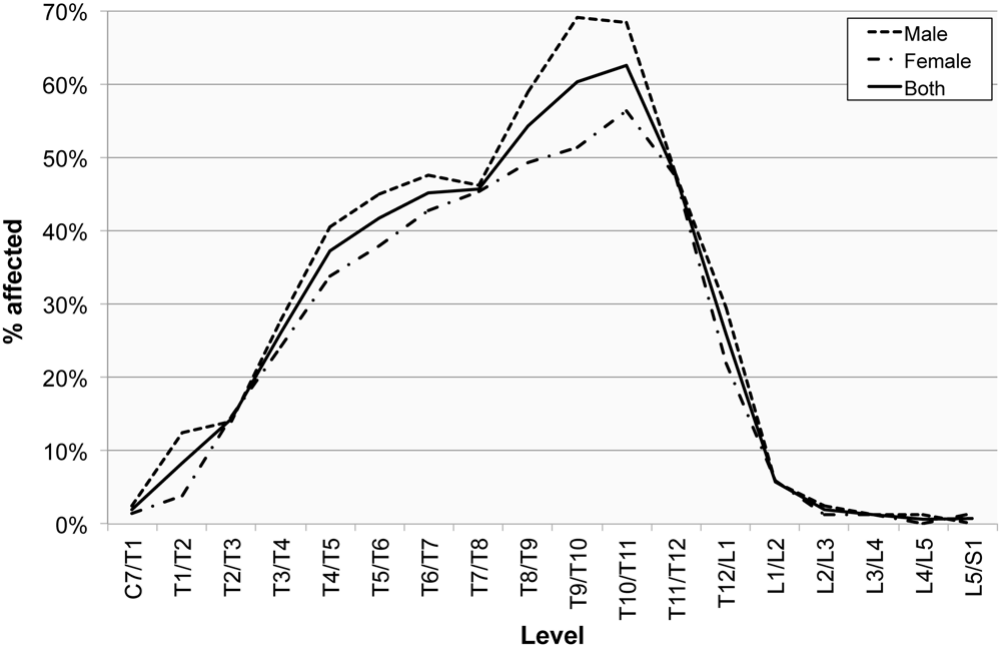
The frequency of ossification of the ligamentum flavum manifested in the spinal levels in adult males (n = 179) and females (n = 166).

Both sexes displayed a similar pattern regarding OLF distribution by level, with no statistically significant difference observed. The peak in OLF manifestation in male spines occurred at the T9/T10 level, which is one level higher than the peak (T10/T11) in female spines. The most noticeable difference in frequencies between the sexes occurred between the T4/T5 and T6/T7, and T8/T9 and T9/T10 levels. A higher frequency in male spines also occurred at the T1/T2 and T12/L1 levels (Fig. 5). Although no statistically significant difference was observed in this study, the difference between OLF levels in males and females in the lower thoracic level may relate to differential loading factors of this part of the spine between males and females. A recent study by Hay *et al*., for instance, has observed a more pronounced lumbar lordosis in females compared to males which they attributed to adaptive factors relating to childbearing^58^.

**Figure 6:**
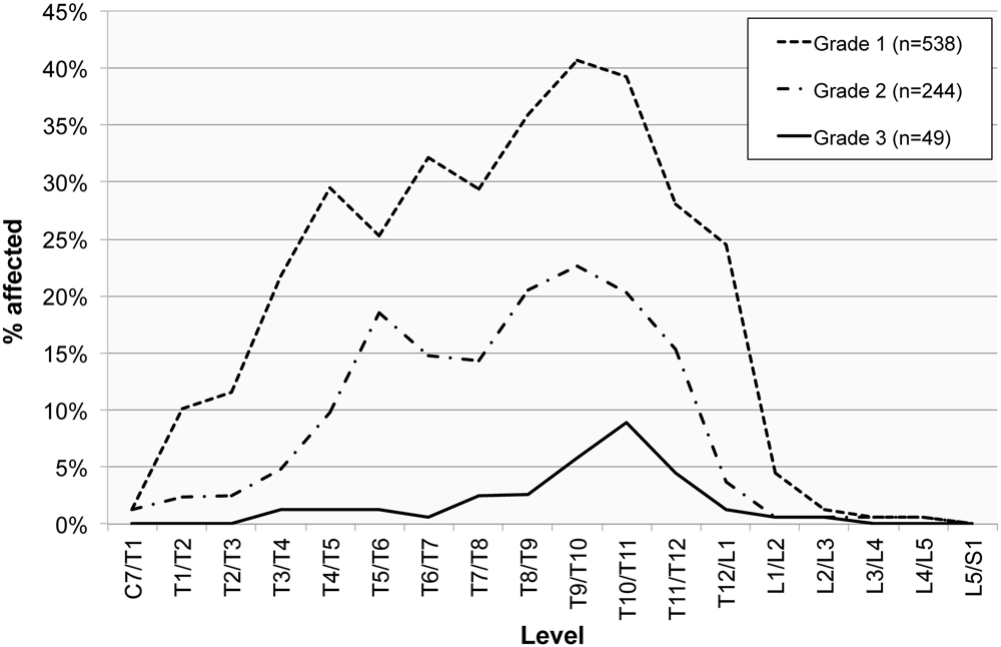
The frequency of ossification of the ligamentum flavum manifested in the spinal levels in males (n = 179) by the degree of ossification (Grade 1 = slight, Grade 2 = moderate; Grade 3 = considerable).

**Figure 7:**
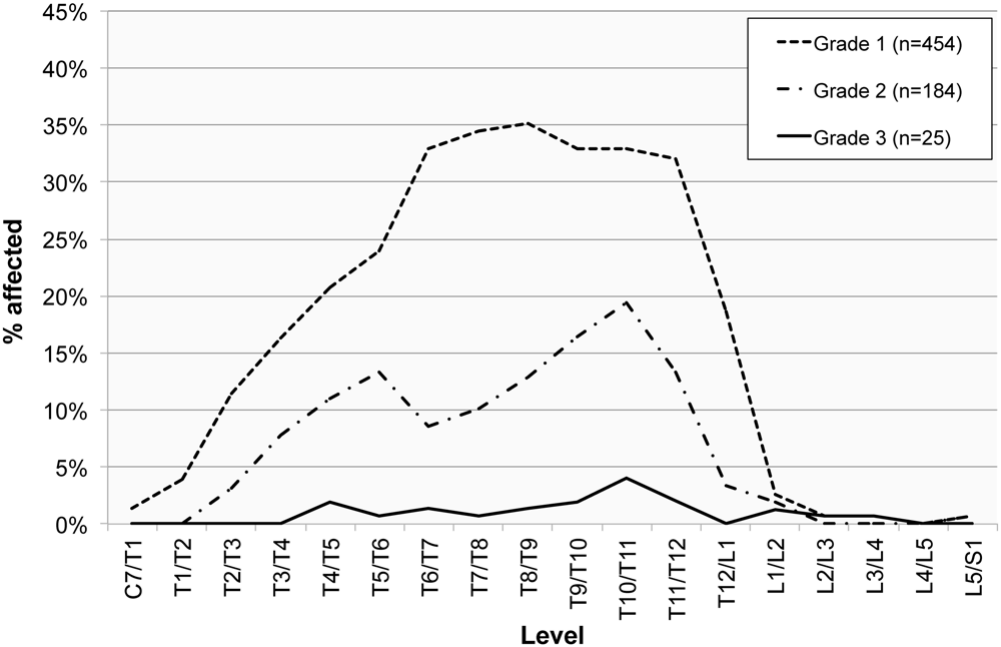
The frequency of ossification of the ligamentum flavum manifested in the spinal levels in adult females (n = 166) by the degree of ossification (Grade 1 = slight, Grade 2 = moderate; Grade 3 = considerable).

When taking the grade of ossification into consideration, males and females display similar patterns. The latter group exhibited a broader peak of slight ossifications (between the T6/T7 and T11/T12 levels) compared to males (with a peak between T9/T10 and T10/T11) (Figs 6 and 7). A statistically significant difference in OLF grades between the sexes, with higher rates in males, only occurred at two levels: at T1/T2 (χ^2^(1) = 8.7, *p* = 0.013) and T9/T10 (χ^2^(1) = 11.7, *p* = 0.009).

### Advancing age determines the prevalence of OLF

**Figure 8:**
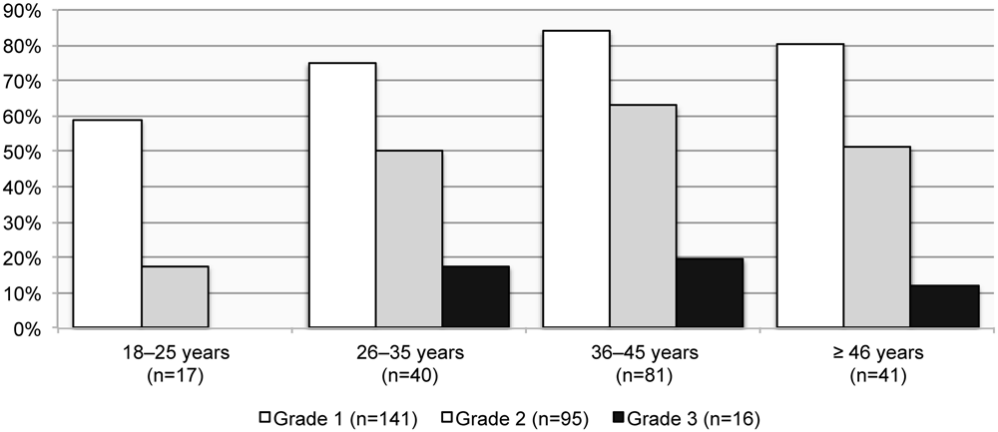
The prevalence of thoracolumbar spines affected by Grade 1 (slight), Grade 2 (moderate) and Grade 3 (considerable) of ossification of the ligamentum flavum manifestations by age group in males.

**Figure 9:**
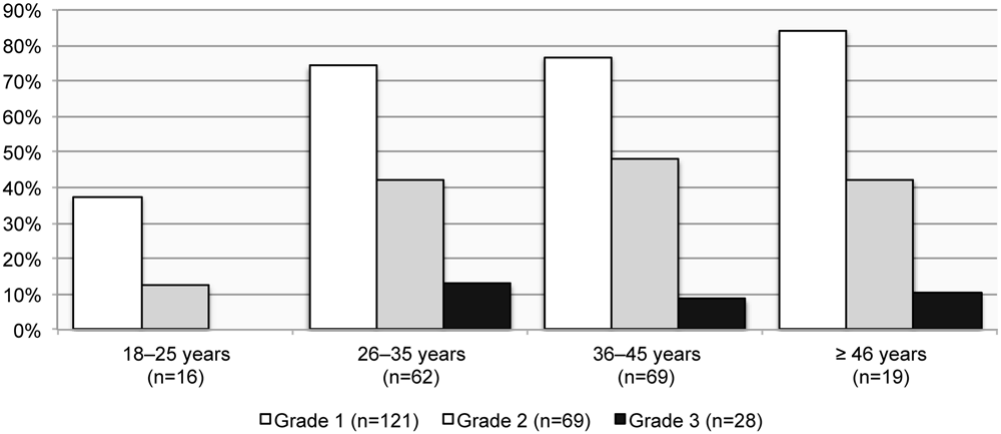
The prevalence of thoracolumbar spines affected by Grade 1 (slight), Grade 2 (moderate) and Grade 3 (considerable) of ossification of the ligamentum flavum manifestations by age group in females.

The prevalence rate of the overall sample is indicating an increase in OLF with advanced age, ranging from 48.5% (16/33) in 18 to 25-year-olds to 83.3% (50/60) in ≥46-year-olds of that population. Grade 1 and Grade 2 ossifications occur in all age groups. Grade 3 ossifications only present in individuals aged over 25 years (Figs 8 and 9). Age was found to be a determining factor, with a medium effect size (*r* = 0.3), for developing OLF in the thoracic region of this sample (see Table 4). No pattern relating to either sex or age was detected when assessing OLF prevalence in the lumbar spine.

Age was a determining factor for OLF Grade 1 and Grade 2 (Table 5), which suggest that OLF Grade 3 was a later progression of the condition that would almost exclusively have occurred in old age (≥46 years).

**Table 5:** Binary logistic regression values of Grade of ossification of the ligamentum flavum by sex (female = 1; male = 2) and age group.

Grade	Predictor	Coeff (SE)	Odds ratio (CI)	*p*-value
0	Constant	6.0 (1.5)	—	<0.001
	Sex	−0.5 (0.3)	0.6 (0.3, 1.1)	0.125
	Age	−0.9 (0.2)	0.4 (0.3, 0.6)	<0.001
1	Constant	−4.5 (1.3)	—	<0.001
	Sex	0.2 (0.3)	1.2 (0.7, 2.1)	0.458
	Age	0.7 (0.2)	2.1 (1.5, 2.9)	<0.001
2	Constant	−4.0 (1.1)	—	<0.001
	Sex	0.4 (0.2)	1.5 (0.9, 2.3)	0.099
	Age	0.4 (0.1)	1.6 (1.2, 2.0)	0.001
3	Constant	−4.2 (1.5)	—	0.006
	Sex	0.5 (0.3)	1.6 (0.9, 3.2)	0.141
	Age	0.2 (0.2)	1.2 (0.8, 1.8)	0.303

CI = confidence interval, Coeff = coefficient, SE = standard error.

### OLF coincides with degenerative spondyloarthropathies

The adult sample from the Kilkenny population revealed five other skeletal spondyloarthropathies in the thoracolumbar spines. Osteoarthritis of the apophyseal joints was diagnosed from the presence of eburnation or combination of articular surface pitting, bony contour change and marginal osteophytes^59^, and was present in 29.3% of females (51/174) and of males 35.1% (65/185). It increased in prevalence with age, ranging from 15.2% (5/33) in 18 to 25-year-olds to 54.8% (34/62) in the ≥46 years age category. Schmorl’s nodes were present in 32.5% (55/169) of females and 53.6% (98/183) of males, ranging from 27.3% (9/33) in 18 to 25-year-olds to 47.5% (28/59) in those aged ≥46 years. Spondylosis deformans was diagnosed in the thoracolumbar spines of 51.1% of females (71/139) and 57.9% of males (81/140) and was observed first in the 26 to 35 years age category at a rate of 32.9% (28/85) which increased to 87.8% (36/51) in ≥46-year-olds. Thoracolumbar intervertebral osteochondrosis was present in 13.0% of females (22/169) and 12.0% of males (22/183), and was observed in 7.6% (8/105) of all 26 to 35-year-olds and increased to 16.9% (10/59) in individuals belonging to the ≥46 years age category. Additionally, five males aged ≥36 years were diagnosed with DISH (diffuse idiopathic skeletal hyperostosis). All conditions, except intervertebral osteochondrosis and DISH in males, correlated in with OLF (Table 6). The results are strongly suggestive that the formation of OLF in this population is due to degenerative wear-and-tear.

**Table 6:** The prevalence rate of ossification of the ligamentum flavum (OLF; by grade and in total) in thoracolumbar spines with spondyloarthropathies present.

Spondyloarthropathy	OLF Grade					χ^2^	*p*-value
	0	1	2	3	1–3		
**Males**							
Osteoarthritis (n = 64)	7.8 (5)	85.9 (55)	54.7 (35)	23.4 (15)	92.2 (59)	5.2	0.039
Schmorl’s nodes (n = 96)	11.5 (11)	86.5 (83)	55.2 (53)	21.9 (21)	88.5 (85)	3.9	0.049
Spondylosis deformans (n = 81)	7.4 (6)	88.9 (72)	64.2 (52)	22.2 (18)	92.6 (75)	8.7	0.003
Intervertebral osteochondrosis (n = 21)	4.8 (1)	90.5 (19)	47.6 (10)	23.8 (5)	95.2 (20)	2.4	0.123
Diffuse idiopathic skeletal hyperostosis (n = 5)	20.0 (1)	80.0 (4)	60.0 (3)	40.0 (2)	80.0 (4)	0.1	0.825
**Females**							
Osteoarthritis (n = 50)	12.0 (6)	84.0 (42)	46.0 (23)	10.0 (5)	88.0 (44)	6.3	0.012
Schmorl’s nodes (n = 53)	11.3 (6)	86.8 (46)	52.8 (28)	17.0 (9)	88.7 (47)	7.3	0.007
Spondylosis deformans (n = 70)	14.3 (10)	84.3 (59)	51.4 (36)	14.3 (10)	85.7 (60)	6.3	0.012
Intervertebral osteochondrosis (n = 22)	4.5 (1)	90.9 (20)	63.6 (14)	13.6 (3)	95.5 (21)	5.4	0.020

Chi-square values are calculated from the OLF Grade 1–3 category (df = 1).

## Discussion

### Is OLF an underreported pathology in people of European ancestry?

Polgár was among the first to report on OLF^34^. His study included radiological examinations and a clinical workup of a 52-year-old male and a 25-year-old female from Hungary, and both cases were affected in the lumbar spine. OLF has so far mainly been described in East Asian populations^1^, which may potentially be related to genetic factors^60,61^. Notable studies have included assessment of Japanese^1,2,5,7,12–19,21–23,26,32^, Chinese^4,8–11,20,25,27–31,62^ and Korean patient samples^63,64^. In these, the prevalence ranges from 3.8% to 26.0%^3,9,63^, though only a fraction required treatment. In these studies, males are more commonly affected than females in both symptomatic^2,65^ and asymptomatic cases^16^. Beyond this, much smaller numbers of OLF have been reported from other ancestral backgrounds, e.g. of Indian^65–68^, African^69–73^ or Arabian^74–76^ descent and rarely any from European ancestral populations^6,77–79^. However, there is some evidence to suggest that OLF might generally be underreported in Europeans^55^. Interestingly, cases have also reported from regions with a mixed European, Arab and East-Asian ancestral background^33^.

Evidence was provided for OLF to exist in a population of European (Irish) ancestry at similar rates as reported previously in East Asians, proving hypothesis A^1,2,5–8,10,11,17,20,24–26,29,31,32,63,74,76,77,80^. Moreover, statistical evidence was found in favour of age progression of OLF, but the trend towards higher rates in males compared to females could not confirmed on a statistical level, rejecting hypothesis B. Comparison of the given archaeological sample to previous clinical publications on the topic indicates that there are notable similarities to contemporary findings on OLF pathology regarding age distribution towards more severe cases and higher prevalence in older age^2,9,11,20,26,31,33,63,71,74^ and sex ratio^2,16,20,26,74,76^ (Table S1 = Age and Gender). Similarly, the most affected levels were in the T10–T12 region^2,3,8,9,11,17,20,22,26,29,31,32,63,71,76^ and a second smaller peak in the upper thoracic region^8,20,31,63^ (Table S2 = Spine levels). There, however, appears to be a large variability regarding the prevalence of OLF^2,3,8,9,11,22,32,63,76^, ranging between 0.5% in an overall group^3^ and 61.5%^22^ in a thoracic myelopathy group. Prevalence rates decreased with increasing sample sizes of the overall patient population^2,3,9,16,22,33,63^ and were the highest in a preselected sample of patients suffering from thoracic myelopathy^22^. It needs to be pointed out that an approach involving patients, as done elsewhere, differs markedly from the approach presented in the given study using an overall population sample; no information on the actual symptoms or myelopathy rates is obtainable, which means that the clinical impact of the OLF rates in this study remains speculative. Additionally, the differential methodological approaches for obtaining data on OLF in clinical (generally based on radiography) and bioarchaeological (based on osteological assessment) studies are too fundamentally different. Yet when including cases of severe ossifications (Grade 3) only, prevalence rates of OLF of 12.8% (44/387) in the Kilkenny sample are similar to rates that may be expected in patients, e.g. as reported by Kudo *et al*.^16^ or Guo *et al*.^9^.

The most comparable investigation of OLF to the given study is Nathan’s macroscopical investigation of what was referred to as ‘para-articular processes’ in the thoracic spines of individuals from the Hamann-Todd Osteological Collection^81^. Nathan observed OLF in 82.1% of Whites (170/207) and 63.8% of Blacks (90/141)^82^. Nathan also observed the generic pattern that is evident in both the Kilkenny sample and reported in the clinical literature, with a peak occurring at the T9 to T11 level^82^. In Nathan’s study, the prevalence of OLF peaked in people aged in their 30s with minute further increase, corroborating the trend given by the Kilkenny data. This implies that the prevalence rates from Kilkenny sample should be considered a representative rate for a mid-nineteenth-century European population. It does also suggest that macroscopic post-mortem investigations may complement radiological studies of OLF, thereby giving a more encompassing view into the pathology of human spines.

### The aetiology and symptomatic impact of OLF is largely unclear

The current understanding of the causes of OLF is uncertain^61^. A number of mechanisms have been proposed. Elastic fibre breakdown^29,62^, collagen proliferation^18,29^ and disarrangement^62^, and apoptotic cell death^18^ are frequently reported in association with OLF. Increased inflammation parameters such as IL-6, TNF-α^26,61^, TGF-β^26,61^, BMP-2 and VEGF^26^, and altered Ca, F, and Zn levels^62^ have also been found in OLF. A recent theory proposes an ossification pathway, via cytokines such as increased IL-1, TNF-α, PG-E_2_ and nitric oxide with elevated osteocalcin, type III and IV collagen expression levels, following disc herniation^35^. It has also been found that tenocytes in the ligamentum flavum are susceptible to adenovirus infections, potentially triggering OLF^62^.

Based on the clinical literature, the onset of OLF-related symptoms with consecutive surgical treatment ranges between 55 and 65 years, as the prevalence increases with age^1–3,6,8,9,32^. Elderly are more likely to be asymptomatic^6^. The results from the Kilkenny sample suggest that the initial onset of OLF occurs in young adulthood, but that formation reaches a peak already at the early middle adult age category (26–35 years), after which it remains relatively constant. The literature has also stressed the influence of intrinsic factors and lifestyle-related effects to the development of OLF, such as the genetic background^1^ and nutrition^60^ of affected individuals, obesity, diabetes, hyperinsulinism and impaired glucose tolerance^22,60^. Similar findings, by Abbas *et al*., have also been reported for lumbar spinal canal stenosis observed clinically, showing that diabetes mellitus in males is related to ligamentum flavum thickening in a significant patient cohort^83^. This study also observed a co-occurrence between ligamentum flavum thickening with consequential spinal canal stenosis and activity patterns (heavy manual labour) in males and body weight (high BMI-values) in females^83^. There are also additional reports on OLF manifestations in relation to fluorosis^29,61,66^, increased bone density^64^, hyperparathyroidism^84^, DISH^61^, ankylosing spondylitis^29,61^, acromegaly^78^ and achondroplasia^21^. External triggers of OLF might likely be increased as a consequence of biomechanical stress^1,15,29^ to the effect of post-traumatic ossification^61,85^. The only condition of the aforementioned examples that were diagnosed in the Kilkenny sample was DISH. Generic ligament ossification is regarded to be a symptom of DISH^86–89^ and is frequently observed in archaeological skeletons diagnosed with the condition^90–92^. In the five cases diagnosed with DISH from the Kilkenny sample, no abnormal ossifications of ligaments were observed. From the data provided here, however, it needs to be stated that no clear extrapolations can be made on the prevalence of OLF in contemporary modern European populations. Nor is it possible to conclude the ratio of individuals that would have been affected by symptoms of OLF from skeletal remains.

### Palaeopathological studies contribute to the understanding of OLF

Palaeopathology—the study of disease in ancient (archaeological) human remains—has a huge potential to add another dimension to the contemporary clinical-medical understanding of disease^93^, as shown in this study. Rogers and Dieppe^88^ highlighted the particular value entailed in the palaeopathological study of rheumatological diseases, stressing its potential to add a vast time dimension to epidemiological studies and the ability to study the earliest osseous onsets of diseases. Another strength of palaeopathology is that it can access large samples, makes population-based studies more nuanced. Similar to the discourse in the clinical disciplines, OLF has rarely been discussed in the bioarchaeological or palaeopathological literature. Some of these few examples include Hukuda *et al*.’s study of ancient Chinese skeletons (dating from 7000 BC to AD 1600), which observed thoracic OLF at crude prevalence rates of 51.7% in males and 29.1% in females^94^. In a study of spondyloarthropathies of a medieval sample (AD 900–1000) from Ostrów Lednicki in Poland, Swedborg observed OLF in 23.6% of male and 15.6% of female thoracic vertebrae^38^. Stirland and Waldron’s study of skeletons from the *Mary Rose* in England (AD 1545) reported frequencies of 46.3% of thoracic and 5.8% of lumbar vertebrae affected by OLF and attributed them physical activity^37^. However, little effort has to date been undertaken to assess OLF in the context of biocultural biomechanics in relation to specific or generic activity patterns in past populations, although this potential has been recognised^95^. To elucidate the potential pathomechanisms further, histology would have been a helpful tool in this assessment. A histological analysis was however not possible to conduct for this current study.

## Conclusions

We have analysed the prevalence and frequency of OLF in the thoracolumbar spines of archaeologically excavated human skeletons from Ireland dating to the mid-nineteenth century. An overall prevalence of 79.7% was observed, and a rate of 12.8% for severe OLF. These findings indicate that OLF is not limited to East-Asian populations, as may be perceived from the published clinical literature. OLF may consequently be largely underreported in populations of European ancestry. Polgár already assumed that given the anatomical location of the ligamentum flavum, its ossification might cause spinal canal stenosis in populations of European ancestry^34^. Future studies are required to validate our findings in a clinical scenario. Additionally, a targeted bioarchaeological study has the potential reveal the influence of sociocultural factors and physical activity patterns to the development of this condition in a long-term historical perspective.

## Electronic supplementary material

Supplementary Tables

## References

[1] Ahn DK (2014). Ossification of the ligamentum flavum. Asian Spine J.

[2] Aizawa T (2006). Thoracic myelopathy in Japan: epidemiological retrospective study in Miyagi Prefecture during 15 years. Tohoku J Exp Med.

[3] Ando K (2013). Predictive factors for a poor surgical outcome with thoracic ossification of the ligamentum flavum by multivariate analysis: a multicenter study. Spine (Phila Pa 1976).

[4] Chen PY, Lin CY, Tzaan WC, Chen HC (2007). Brown-Sequard syndrome caused by ossification of the ligamentum flavum. J Clin Neurosci.

[5] Enomoto H, Kuwayama N, Katsumata T, Doi T (1988). Ossification of the ligamentum flavum: a case report and its MRI finding. Neuroradiology.

[6] Fotakopoulos G, Alexiou GA, Mihos E, Voulgaris S (2010). Ossification of the ligamentum flavum in cervical and thoracic spine: report of three cases. Acta Neurol Belg.

[7] Fujimoto K (2015). Neurologic findings caused by ossification of ligamentum flavum at the thoracolumbar junction. J Spinal Cord Med.

[8] Gao R, Yuan W, Yang L, Shi G, Jia L (2013). Clinical features and surgical outcomes of patients with thoracic myelopathy caused by multilevel ossification of the ligamentum flavum. Spine J.

[9] Guo JJ, Luk KD, Karppinen J, Yang H, Cheung KM (2010). Prevalence, distribution, and morphology of ossification of the ligamentum flavum: a population study of one thousand seven hundred thirty-six magnetic resonance imaging scans. Spine (Phila Pa 1976).

[10] Hasue M (1980). Roentgenographic analysis of ossification of the spinal ligament with special reference to the finding of the whole spine. Seikei Geka.

[11] He S, Hussain N, Li S, Hou T (2005). Clinical and prognostic analysis of ossified ligamentum flavum in a Chinese population. J Neurosurg Spine.

[12] Hirai T, Korogi Y, Takahashi M, Shimomura O (2001). Ossification of the posterior longitudinal ligament and ligamentum flavum: imaging features. Semin Musculoskelet Radiol.

[13] Inoue H, Seichi A, Kimura A, Endo T, Hoshino Y (2013). Multiple-level ossification of the ligamentum flavum in the cervical spine combined with calcification of the cervical ligamentum flavum and posterior atlanto-axial membrane. Eur Spine J.

[14] Kim K, Isu T, Nomura R, Kobayashi S, Teramoto A (2008). Cervical ligamentum flavum ossification: two case reports. Neurol Med Chir (Tokyo).

[15] Kotani Y (2013). Cervical myelopathy resulting from combined ossification of the ligamentum flavum and posterior longitudinal ligament: report of two cases and literature review. Spine J.

[16] Kudo S, Ono M, Russell WJ (1983). Ossification of thoracic ligamenta flava. Am J Roentgenol.

[17] Matsumoto Y (2012). Clinical characteristics and surgical outcome of the symptomatic ossification of ligamentum flavum at the thoracic level with combined lumbar spinal stenosis. Arch Orthop Trauma Surg.

[18] Nakama S (2005). Regional difference in the appearance of apoptotic cell death in the ligamentum flavum of the human cervical spine. Med Mol Morphol.

[19] Okuda T (2004). The pathology of ligamentum flavum in degenerative lumbar disease. Spine (Phila Pa 1976).

[20] Sun J (2014). Surgical strategies for ossified ligamentum flavum associated with dural ossification in thoracic spinal stenosis. J Clin Neurosci.

[21] Suzuki K, Kanamori M, Nobukiyo M (2008). Ossification of the thoracic ligamentum flavum in an achondroplastic patient: a case report. J Orthop Surg (Hong Kong).

[22] Takenaka S (2014). Neurological manifestations of thoracic myelopathy. Arch Orthop Trauma Surg.

[23] Takeuchi Y (1989). High incidence of obesity and elevated serum immunoreactive insulin level in patients with paravertebral ligamentous ossification: a relationship to the development of ectopic ossification. J Bone Miner Metab.

[24] Wang ZL, Yuan HF, Ding HQ, Zhao HN, Qiao YD (2006). [The clinical causes of the thoracic ossification of ligamentum flavum]. Zhonghua Wai Ke Za Zhi.

[25] Xiong L, Zeng QY, Jinkins JR (2001). CT and MRI characteristics of ossification of the ligamenta flava in the thoracic spine. Eur Radiol.

[26] Yayama T (2007). Thoracic ossification of the human ligamentum flavum: histopathological and immunohistochemical findings around the ossified lesion. J Neurosurg Spine.

[27] Feng F, Sun C, Chen Z (2015). A diagnostic study of thoracic myelopathy due to ossification of ligamentum flavum. Eur Spine J.

[28] Feng FB, Sun CG, Chen ZQ (2015). Progress on clinical characteristics and identification of location of thoracic ossification of the ligamentum flavum. Orthop Surg.

[29] Li F, Chen Q, Xu K (2006). Surgical treatment of 40 patients with thoracic ossification of the ligamentum flavum. J Neurosurg Spine.

[30] Li M (2012). Management of thoracic myelopathy caused by ossification of the posterior longitudinal ligament combined with ossification of the ligamentum flavum-a retrospective study. Spine J.

[31] Li WJ, Guo SG, Sun ZJ, Zhao Y (2015). Multilevel thoracic ossification of ligamentum flavum coexisted with/without lumbar spinal stenosis: staged surgical strategy and clinical outcomes. BMC Musculoskelet Disord.

[32] Kawaguchi Y (2016). Characteristics of ossification of the spinal ligament; incidence of ossification of the ligamentum flavum in patients with cervical ossification of the posterior longitudinal ligament: analysis of the whole spine using multidetector CT. J Orthop Sci.

[33] Ergun T, Lakadamyali H (2012). The relationship between the prevalance and size of lumbar ossified ligamentum flavum and the presence and degree of facet joint degeneration. Eur J Radiol.

[34] Polgár F (1920). Über interarkuelle Wirbelverkalkung. Fortschr Geb Roentgenstr.

[35] Kang YM (2014). Herniated intervertebral disk induces hypertrophy and ossification of ligamentum flavum. J Spinal Disord Tech.

[36] Waldron, T. *Palaeopathology*. (Cambridge University Press, 2009).

[37] Stirland AJ, Waldron T (1997). Evidence of activity related markers in the vertebrae of the crew of the Mary Rose. J Archaeol Sci.

[38] Swedborg, I. *Degenerative changes of the human spine: a study on dried macerated skeletons*. (PhD thesis, University of Stockholm, 1974).

[39] Paja L (2010). Diffuse idiopathic skeletal hyperostosis: appearance and diagnostics in Hungarian osteoarchaeological materials. Acta Biol Szeged.

[40] Geber, J. *Victims of Ireland’s Great Famine: the bioarchaeology of mass burials at Kilkenny Union Workhouse*. (University Press of Florida, 2015).

[41] Kinealy, C. *This great calamity: the Irish Famine 1845–52*. 2nd edn, (Gill & Macmillan, 2006).

[42] Crowley, J., Smyth, W. J. & Murphy, M. eds *Atlas of the Great Irish Famine*, 1845–52. (Cork University Press, 2012).

[43] Geber J (2016). Mortality among institutionalised children during the Great Famine in Ireland: bioarchaeological contextualisation of non-adult mortality rates in the Kilkenny Union Workhouse, 1846–1851. Contin Chang.

[44] Keenan, D. *Pre-famine Ireland: Social structure*. (Xlibris Corporation, 2000).

[45] Brickley, M. & McKinley, J. I. eds *Guidelines to the standards for recording human remains*. (British Association for Biological Anthropology and Osteoarchaeology/Institute of Field Archaeologists, 2004).

[46] Buikstra, J. E. & Ubelaker, D. H. *Standards for data collection from human skeletal remains: proceedings of a seminar at the Field Museum of Natural History*. (Arkansas Archeological Survey Press, 1994).

[47] Sjøvold, T. In *Anthropologie: Handbuch der vergleichenden Biologie des Menschen. Band I: Wesen und Methoden der Anthropologie. 1. Teil: Wissenschaftstheorie, Geschichte, morphologische Methoden* (ed. Rainer Knußmann) 444–480 (Gustav Fischer Verlag, 1988).

[48] Lovejoy CO, Meindl RS, Pryzbeck TR, Mensforth RP (1985). Chronological metamorphosis of the auricular surface of the ilium: a new method for the determination of adult skeletal age at death. Am J Phys Anthropol.

[49] İşcan MY, Loth SR (1986). Determination of age from the sternal rib in white Females: a test of the phase method. Journal of Forensic Sciences.

[50] İşcan MY, Loth SR, Wright RK (1984). Metamorphosis at the sternal rib end: a new method to estimate age at death in white males. Am J Phys Anthropol.

[51] Meindl RS, Lovejoy CO (1985). Ectocranial suture closure: A revised method for the determination of skeletal age at death based on the lateral-anterior sutures. American Journal of Physical Anthropology.

[52] Brooks S, Suchey JM (1990). Skeletal age determination based on the os pubis: A comparison of the Acsádi-Nemeskéri and Suchey-Brooks methods. Hum Evol.

[53] Cox, M. In *Human osteology in archaeology and forensic science* (eds Margaret Cox & Simon Mays) 61–81 (Cambridge University Press, 2000).

[54] McKinley, J. I. In *Guidelines to the standards for recording human remains IFA Paper* (eds Megan Brickley & Jacqueline I. McKinley) 14–20 (British Association for Biological Anthropology and Osteoarchaeology/Institute of Field Archaeologists, 2004).

[55] Williams DM, Gabrielsen TO, Latack JT, Martel W, Knake JE (1984). Ossification in the cephalic attachment of the ligamentum flavum: an anatomical and CT study. Radiology.

[56] Williams DM, Gabrielsen TO, Latack JT (1982). Ossification in the caudal attachments of the ligamentum flavum. Radiology.

[57] Powers, N. ed. *Human osteology method statement*. (Museum of London, 2012).

[58] Hay O (2015). The lumbar lordosis in males and females, revisited. Plos One.

[59] Rogers, J. & Waldron, T. *A field guide to joint disease in archaeology*. (John Wiley & Sons, 1995).

[60] Mobbs RJ, Dvorak M (2007). Ossification of the ligamentum flavum: diet and genetics. J Clin Neurosci.

[61] Ren L (2013). The roles of inflammatory cytokines in the pathogenesis of ossification of ligamentum flavum. Am J Transl Res.

[62] Wang Z, Li XD, Li MQ, Wang QP (2008). Changes in basic metabolic elements associated with the degeneration and ossification of ligamenta flava. J Spinal Cord Med.

[63] Moon BJ (2015). Prevalence, distribution, and significance of incidental thoracic ossification of the ligamentum flavum in Korean patients with back or leg Pain: MR-based cross sectional study. J Korean Neurosurg Soc.

[64] Sohn S, Yoon JW, Chung CK (2014). Increased bone mineral density in patients with ossification of the ligamentum flavum: a case-control study. J Clin Densitom.

[65] Hussain M, Ahmed Raja R, Makhdoom A (2014). Ossification and hypertrophy of ligamentum flavum at thoracic spine. J Ayub Med Coll Abbottabad.

[66] Gupta RK (1996). Compressive myelopathy in fluorosis: MRI. Neuroradiology.

[67] Muthukumar N (2009). Dural ossification in ossification of the ligamentum flavum: a preliminary report. Spine (Phila Pa 1976).

[68] Nadkarni TD, Menon RK, Desai KI, Goel A (2005). Ossified ligamentum flavum of the atlantoaxial region. J Clin Neurosci.

[69] Cabre P (2001). Six cases of cervical ligamentum flavum calcification in Blacks in the French West Indies. Joint Bone Spine.

[70] Jaffan I, Abu-Serieh B, Duprez T, Cosnard G, Raftopoulos C (2006). Unusual CT/MR features of putative ligamentum flavum ossification in a North African woman. Br J Radiol.

[71] Pascal-Moussellard H, Cabre P, Smadja D, Catonne Y (2005). Symptomatic ossification of the ligamentum flavum: a clinical series from the French Antilles. Spine (Phila Pa 1976).

[72] Shepard NA, Shenoy K, Cho W, A DS (2015). Extensive ossification of the ligamentum flavum treated with triple stage decompression: a case report. Spine J.

[73] Suojanen JN, Lipson SJ (1989). Spinal cord compression secondary to ossified ligamentum flavum. J Spinal Disord.

[74] Al-Jarallah K, Al-Saeed O, Shehab D, Dashti K, Sheikh M (2012). Ossification of ligamentum flavum in Middle East Arabs: a hospital-based study. Med Princ Pract.

[75] al-Orainy IA, Kolawole T (1998). Ossification of the ligament flavum. Eur J Radiol.

[76] Ben Hamouda K, Jemel H, Haouet S, Khaldi M (2003). Thoracic myelopathy caused by ossification of the ligamentum flavum: a report of 18 cases. J Neurosurg.

[77] Goodman JM, Kuzma BB (1996). Ossification of the ligamentum flavum with myelopathy. Surg Neurol.

[78] Schmidt RF, Goldstein IM, Liu JK (2013). Ossified ligamentum flavum causing spinal cord compression in a patient with acromegaly. J Clin Neurosci.

[79] Shiraishi T, Crock HV, Lewis P (1995). Thoracic myelopathy due to isolated ossification of the ligamentum flavum. J Bone Joint Surg Br.

[80] Yabe Y (2013). Thoracic radiculopathy caused by ossification of the ligamentum flavum. Ups J Med Sci.

[81] Kern KFT (2006). Wingate Todd: Pioneer of Modern American Physical Anthropology. Kirtlandia.

[82] Nathan H (1959). The para-articular processes of the thoracic vertebrae. Anat Rec.

[83] Abbas J (2013). Socioeconomic and physical characteristics of individuals with degenerative lumbar spinal stenosis. Spine.

[84] Sabanis, N. *et al*. Rare skeletal complications in the setting of primary hyperparathyroidism. *Case Rep Endocrinol* **2015**, 10.1155/2015/139751 (2015). 10.1155/2015/139751 PMC4664786 26664767

[85] Costa F, Perotti F (1951). Posttraumatic ossification of intervertebral ligamenta flava. Arch Ortop.

[86] Cammisa M, De Serio A, Guglielmi G (1998). Diffuse idiopathic skeletal hyperostosis. European Journal of Radiology.

[87] Resnick D, Shaul SR, Robins JM (1975). Diffuse Idiopathic Skeletal Hyperostosis (DISH): Forestier’s Disease with Extraspinal Manifestations. Radiology.

[88] Rogers J, Dieppe P (1990). Skeletal palaeopathology and the rheumatic diseases: where are we now?. Ann Rheum Dis.

[89] Rogers J, Waldron T, Dieppe P, Watt I (1987). Arthropathies in palaeopathology: the basis of classification according to most probable cause. J Archaeol Sci.

[90] Verlaan JJ, Oner FC, Maat GJR (2007). Diffuse idiopathic skeletal hyperostosis in ancient clergymen. Eur Spine J.

[91] Suzuki T, Fujita H, Narasaki S, Kondo O, Adachi K (1993). A study of skeletal remains with diffuse idiopathic skeletal hyperostosis (DISH) from the Edo period, Japan. Anthropol Sci.

[92] Bernert Z (2003). Two cases of arthritis disease from the medieval age, Hungary. Anthropol.

[93] Dieppe P, Loe L, Shepstone L, Watt I (2006). What ‘skeletal paleopathology’ can teach us about arthritis: The contributions of the late Dr Juliet Rogers. Reumatismo.

[94] Hukuda S (2000). Spinal degenerative lesions and spinal ligamentous ossifications in ancient Chinese populations of the Yellow River civilization. Int J Osteoarchaeol.

[95] Villotte S (2006). Connaissances médicales actuelles, cotation des enthésopathies: nouvelle méthode. Bull Mém Soc Anthropol Paris.

