# Supplementary material for: Ossification of the Ligamentum Flavum in a Nineteenth-Century Skeletal Population Sample from Ireland: Using Bioarchaeology to Reveal a Neglected Spine Pathology
Source: Sci Rep. 2018 Jun 18;8:9313. doi: 10.1038/s41598-018-27522-x (PMC6006438; doi:10.1038/s41598-018-27522-x)
Supplement: Supplementary file 1 — Supplementary Tables [file 41598_2018_27522_MOESM1_ESM.pdf]

Title (141 characters):

Ossification of the Ligamentum Flavum in a Nineteenth-Century Skeletal Population Sample from Ireland: Using Bioarchaeology to Reveal a Neglected Spine Pathology

Short title (38 characters):

Ossification of the Ligamentum Flavum (OLF)

Author list:

Jonny Geber [1], Niels Hammer [1,2]

Affiliation:

1 Department of Anatomy, University of Otago, Dunedin, New Zealand

2 Department of Orthopaedic and Trauma Surgery, University of Leipzig, Leipzig, Germany

Corresponding authors:

Jonny Geber and Niels Hammer, Department of Anatomy, University of Otago, Lindo Ferguson Building, 270 Great King St, Dunedin 9016, New Zealand; Phone: +64 3 479 7349, Fax: +64 3 479 7254, Email: jonny.geber@otago.ac.nz, nlshammer@googlemail.com

Author contributions:

J.G. undertook the osteological analysis and recording of the data in the study sample. J.G. and N.H. have thereafter contributed equally to the study design, data analysis, and interpretation of the results and drafting of the text.

Word count: 5,183 (excluding abstract and references)

Number of figures: 9

Number of tables: 6

Number of supplementary tables: 2

Table S1 Distribution of ossification of the ligamentum flavum (OLF) over the different age ranges (years) and gender.

Study	Cases with OLF	Gender	0–29	30–39	40–49	50–59	60–69	70–79	≥80
[1]	264	both	1	15	33	66	94	50	5
	80	♀		3	8	20	25	20	4
	184	♂	1	12	25	46	69	30	1
[9]	86	both		2	24	60			
	72	♀	0	2	18	52			
	14	♂	0	0	6	8			
[11]	16	both			3	5	6	2	
	5	♀				1	3	1	
	11	♂			3	4	3	1	
[20]	33	both		1	3	20	5	4	
	15	♀		1		10	3	1	
	18	♂			3	10	2	3	
[26]	43	both		1	3	14	13	12	
	22	♀		1	1	7	7	6	
	21	♂			2	7	6	6	
[31]	?	both		3	1	5	2		
	?	♀			1	4	2		
	?	♂		3		1			
[33]	40	both	4	5	7	7	9	8	
[62]	?	both	4	8	22	72	130	107	7
[68]	6	both					1	5	
	5	♀					1	4	
	1	♂						1	
[70]	14	both		1	1		4	8	
	8	♀		1	1		2	4	
	6	♂					2	4	
[73]	19	both		2	5	7	4	1	
	8	♀			4	3	1		
	11	♂		2	1	4	3	1	
[75]	18	both		1	7	4	4	2	
	3	♀		1	1	1			
	15	♂			6	3	4	2	

Table S2 Distribution of ossification of the ligamentum flavum (OLF) over the different vertebral levels. The highest frequencies of the subsamples are indicated in bold. Two marked peaks can be found for OLF in the upper and lower thoracic spine. *sic

Study	Origin	Sample	Number affected with OLF	Prevalence	C3/4	C4/5	C5/6	C6/7	C7/T1	T1/2	T2/3	T3/4	T4/5	T5/6	T6/7	T7/8	T8/9	T9/10	T10/11	T11/12	T12/L1	L1/2	L2/3	L3/4	L4/5	L5/S1
[1]	Japanese	14,458 surgery patients	219	1.50%					1%	1%	8%	4%	1%	1%	1%	1%	4%	11%	28%	36%	4%					
[3]	Japanese	19,364 with spinal surgery	96	0.50%						9%	3%	88%														
[8]	Chinese		75						7%	9%	37%	33%	37%	33%	32%	31%	40%	63%	72%	60%	21%					
[9]	Chinese	1,736 volunteers	66	3.80%		2%	5%			6%	11%	12%	8%	8%	6%	8%	8%	24%	38%	9%	2%	2%				
[11]	Chinese		27							11%	15%	15%	4%	4%	7%	7%	7%	7%	41%	37%	7%					
[16]	Japanese	1,744 unspecified patients		6.2% ♂, 4.8% ♀																						
[17]	Japanese		36								3%	3%	6%		3%		22%	22%	47%	56%						
[20]	Chinese		33								9%	6%	9%	3%	12%	18%	9%	24%	41%	26%	3%	3%				
[22]	Japanese	205 thoracic myelopathy patients	126	61.50%					2%	3%	11%	8%	6%	4%	6%	6%	9%	25%	50%	40%	2%	2%				
[26]	Japanese		43								2%	12%	21%	16%	2%	5%	19%	26%	53%	44%	14%					
[29]	Chinese		40							15%	25%	15%	5%		10%	10%	15%	75%	105%*	90%	55%					
[30]	Chinese		11							27%	36%	55%	55%	55%	55%	36%	55%	82%	73%	45%	18%	9%	27%	36%	36%	
[32]	Japanese	178 patients with ossification of the posterior longitudinal ligament	116		1%				2%	8%	21%	27%	25%	14%	9%	7%	7%	17%	19%	15%	8%	8%	4%	4%	1%	
[33]	Turkey	114 urologic patients	40	35.10%																		48%	70%	40%	15%	13%
[62]	Korean	2,134 back pain patients	360	16.90%						2%	5%	8%	5%	4%	4%	6%	16%	35%	58%	27%	2%					
[68]	African Caribbean		6		33%	50%	33%	17%																		
[70]	African Caribbean		14						21%	7%	14%	21%	14%					14%	57%	43%						
[73]	Arabs		19			16%	42%						5%		5%		16%		11%						16%	
[74]	Arabs	82 patients with computed tomography	29	35.40%					14%										3%	3%	10%	14%	7%	66%	24%	28%
[75]	Arabs	patients with thoracic myelopathy	18							6%	17%			6%	17%			6%	28%	28%	6%					
